# A machine learning-based clinical predictive tool to identify patients at high risk of medication errors

**DOI:** 10.1038/s41598-024-83631-w

**Published:** 2024-12-30

**Authors:** Ammar Abdo, Lyse Gallay, Thibault Vallecillo, Justine Clarenne, Pauline Quillet, Vincent Vuiblet, Rudy Merieux

**Affiliations:** 1 Institut d’Intelligence Artificielle en Santé, CHU de Reims, Université de Reims Champagne- Ardenne, Reims, F-51100 France; 2 Department of Pharmacy, CHU de Reims, Reims, F-51100 France

**Keywords:** Machine learning, Prediction model, Medication errors, Medication reconciliation, Patient safety, Health care, Medical research, Predictive markers, Machine learning, Drug safety, Risk factors

## Abstract

A medication error is an inadvertent failure in the drug therapy process that can cause serious harm to patients by increasing morbidity and mortality and are associated with significant economic costs to the healthcare system. Medication reconciliation is the most cost-effective intervention and can result in a 66% reduction in medication errors. To improve patient safety, we developed a machine learning-based tool that prioritizes patients at risk of medication errors upon admission to the hospital to ensure that they undergo medication reconciliation by clinical pharmacists. The data were collected from the electronic health records of patients admitted to Reims University Hospital who underwent medication reconciliation between 2017 and 2023. The data from 7200 patients were used to train four machine learning-based models based on 52 variables in the development dataset. These models were used to prioritize admitted patients according to their likelihood of being exposed to a medication error. Our models, particularly the voting classifier model, demonstrated good performance (a recall of 0.75, precision of 0.65, F1 score of 0.70, AUROC of 0.74 and AUCPR of 0.75). In a retrospective evaluation simulating real-life use, the voting classifier model successfully identified 45% of the total patients selected who were found to have at least one unintended discrepancy compared to 21% when using the existing tool. The positive experimental results of this tool showed a superior improvement of 113% over the existing tool by targeting patients at risk of medication errors upon admission to Reims University Hospital.

## Introduction

A medication error (ME) is an unintentional discrepancy in the medication treatment process. Unintentional discrepancies occur when a prescriber unintentionally changes, adds, or omits a medication that a patient was taking prior to admission to the hospital, resulting in or likely to result in harm to the patient^1^. Errors in prescribing, dispensing, drug duplication, dosing errors, storing, preparing, and administering medication are the most common preventable causes of unwanted adverse events in the practice of medication. It can occur at any time (most cases occurred on the first day of hospitalization)^2^. It can occur throughout the patient’s care pathway and can result in a risk or an adverse event for the patient^3^. However, approximately half of adverse drug events are preventable medication errors^4^.

There is potential for many medication errors to occur due to the lack of communication between health professionals, insufficient patient information on how to take their medicine, polymedication of the patient and the risk of confusion between his or her different treatments and inadequate information or packaging of the medicine. These MEs can cause an increase in morbidity and mortality, are associated with a significant economic cost and are estimated to cost US$42 billion annually worldwide^5^. This figure is 0.7% of all global health spending. MEs are the third leading cause of death in the United States and Europe, surpassed only by heart disease and cancer^6^. Among the various actions taken to prevent the occurrence of these MEs is medication reconciliation (MR). MR refers to the process of avoiding such medication discrepancies across transitions in care (admission, transfer, and discharge) by creating as complete and accurate a list as possible of a patient’s current medications and comparing the list to those in the patient’s record or medication orders^7^. MR is the most cost-effective intervention^8^ and can result in a 66% reduction in medication errors^9^.

Currently, MR is flourishing increasingly in France due to the favorable regulatory environment. In fact, since 2016, a decree has been issued to make clinical pharmacy a compulsory activity for internal use^10^. In addition, since January 2018, MR has been an indicator of contracts for improving the quality and efficiency of care and has been a binding contract between regional health agencies and health institutions^11^. Many studies have demonstrated the benefit of MR on patient admission^12–14^. Moreover, in France, MR at patient admission has been developed in many hospitals and, more specifically, in clinical departments such as surgery^15,16^, geriatrics^17–19^, emergencies^20^ and internal medicine departments^21,22^.

At the University Hospital Center (CHU) of Reims, MR is carried out retroactively upon admission to the hospital by 5th -year pharmacy students present in care services. This pharmaceutical activity has been an integral part of their mission since 2014. Students were pretrained on this activity at the College of Pharmacy and were accompanied by a reference pharmacist from the care service. MR is carried out according to a process consisting of 3 stages: actively seeking information on the patient’s healthcare products, carrying out the medication review, and updating the prescription and patient records. However, MR on admission is a time-consuming and expensive process that presents a hospital-wide challenge. MR takes an average of 50 min per patient^7^, necessitating the deployment of more clinical pharmacists, which many healthcare facilities cannot afford and are unwilling to do. Many previous studies have investigated variables that influence pharmaceutical interventions and can be used as predictors^16,23,24^. Other studies have discussed developing a score to identify patients at risk of medication errors using a multivariate logistic regression model^25–27^, but to the best of the authors’ knowledge, none have focused on machine learning-based techniques with patients admitted to different hospital wards, making this paper a novel contribution to this field.

At present, at the CHU, no instructions are given to pharmacy students performing MR regarding patient selection. Medication reconciliations are carried out on adult patients admitted to medical, surgical and obstetric wards, as well as in follow-up care and rehabilitation. These procedures were carried out within 72 h of patient admission. Owing to the human resources allocated, it is not possible to carry out MR for all patients admitted to our institution. To increase the efficiency of this activity, we need to be able to identify those patients most at risk of unintentional discrepancies and prioritize the MR of these patients. At the CHU of Reims, we used the software Easily version 7.0.0.13 to computerize the care files for patients. Easily covers a wide range of functionalities, including the visualization of laboratory results, clinical files, care files and prescriptions and the management of medical letters, including digital dictation and the management of appointments. However, at present, no study has identified patients at risk of MEs upon hospitalization at the entire CHU of Reims. Currently, the increase in the number of electronic health records and the development of big data analytics are paving the way for the use of artificial intelligence (AI) and machine learning (ML) techniques to process these data, which makes MR more effective and helps reduce MEs^28^. Therefore, the aim of this study was to develop a machine learning-based tool to assist clinical pharmacists in selecting patients at high risk of medication errors for medication reconciliation. This approach could improve patient safety, lead to a more efficient process, prevent medication errors, reduce disease complications, and reduce the cost and burden on the healthcare system.

## Materials and methods

### Data collection

The prediction tool developed in this study was derived based on the data of patients admitted to the CHU (Reims, France). CHU is a 2,382-bed general hospital that receives approximately 93,000 clinical admissions each year and provides both surgical and medical services. In the CHU, all patient files are typically digitized and recorded using Easily software (except for neonatology and intensive care units). Prescription orders and MRs by clinical pharmacists are managed with the same software, which includes all patient details, medical and nursing notes, laboratory results, and vital signs.

Only patients who received MR between 2017 and 2023 were considered. MR consists of a standardized medication interview with the patient conducted by a practitioner (pharmacist or pharmacist-in-training). The data were extracted directly from the Easily software database. In addition, because MR sheets are often poorly linked to the respective hospitalizations in Easily software, relinking was performed using patient identifiers, hospitalization dates, and MR dates (considered reliable information). MRs that could not be associated with hospitalization were excluded. The final cohort was validated in consultation with pharmacists. Throughout this study, all the data were stored on a secure local server, and pseudonymous patient identities were used instead of their names (an identification number for each patient).

### Data preprocessing

Data preprocessing is the first step in the ML algorithm and is used to transform the raw data into useful, understandable and efficient formats. Out of 350,540 patients admitted to the hospital between 2017 and 2023, only 12,604 patients were randomly selected for MR, with a total of 52 variables representing each patient’s record.

In this study, the first step in data preprocessing was to improve the data quality, mainly by rectifying outlier values and by filling in possible empty values. To this end, each incorrect or empty numerical value (excluding counters) was filled with the mean of the associated column, while the counter and Boolean values were filled with zeros. In addition, checks on uniqueness (particularly at the level of identifying variables) and correct ordering (particularly at the level of dates, before conversion into numeric variables) were carried out. The second step was data normalization, which was used to adjust the values of variables in the dataset to a common scale. Here, min–max normalization was used where all variable values ranged between [0,1].

The final dataset used included 4361 negative patients and 2839 positive patients. Here, those who were found to have unintended medication discrepancies during medication reconciliation are referred to as positive patients, while those who had no unintended discrepancies are referred to as negative patients. To determine whether handling balanced data contributes to improved performance, training datasets were prepared with/without resampling. The preprocessed dataset was undersampled by the edited nearest neighbor (ENN) method^29^ and then oversampled by adaptive synthetic sampling (ADASYN)^30^. The ENN and ADASYN algorithms were applied using the imbalanced-learn library^31^ in the Python 3.9 language with the default settings.

**Table 1:** Baseline patient characteristics.

	Total (*n* = 7200)	No MEs (*n* = 4361)	MEs (*n* = 2839)	*p* value
Sociodemographic: State at the moment of the MR:				
Age, years	73 (std 15.5)	73 (std 16.2)	73 (std 14.1)	0.604
Sex, male	3288 (46%)	1977 (45.3%)	1311 (46.2%)	0.497
Body mass index (BMI)	24 (std 7.5)	24.2 (std 7.9)	24.8 (std 6.9)	**0.002**
^*^Patient lives in				
Home	5757 (80%)	3634 (83.3%)	2123 (74.8%)	**< 0.0001**
Institution	21 (0.3%)	16 (0.4%)	5 (0.2%)	0.214
Hospitalization: State at the moment of the admission:				
Admission during vacation period	1777 (24.7%)	1068 (24.5%)	709 (25.0%)	0.662
Admission during week-end	1221 (17.0%)	672 (15.4%)	549 (19.3%)	**< 0.0001**
Admission during				
Preshift (5:30 pm to 6 pm or 8 am to 8:30 am)	358 (5.0%)	215 (4.9%)	143 (5.0%)	0.882
Night shift (6 pm to 8 am)	2212 (30.7%)	1281 (29.4%)	931 (32.8%)	**0.002**
Admission from emergency unit	2963 (41.2%)	1924 (44.1%)	1039 (36.6%)	**< 0.0001**
Admission in emergency unit within the past 30 days	1012 (14.1%)	710 (16.3%)	302 (10.6%)	**< 0.0001**
Previous hospitalization within the past 30 days	1063 (14.8%)	696 (16.0%)	367 (12.9%)	**0.0004**
Previous hospitalization within the past 6 months	2478 (34.4%)	1527 (35.0%)	951 (33.5%)	0.194
First hospitalization	2769 (38.5%)	1631 (37.4%)	1138 (40.1%)	0.024
Number of doctors associated with the patient	3 (std 3.0)	3 (std 3.0)	3 (std 2.9)	**< 0.0001**
State at the moment of the MR:				
Occupancy rate of the ward	0.98 (std 0.2)	0.87 (std 0.2)	0.87 (std 0.2)	0.888
Hospitalization in surgical unit	2525 (35.1%)	1275 (29.2%)	1250 (44.0%)	**< 0.0001**
Hospitalization in rehabilitation unit	732 (10.2%)	542 (12.4%)	190 (6.7%)	**< 0.0001**
Time elapsed since the admission (hours)	107 (std 308.1)	120 (std 376.8)	88 (std 148.7)	**< 0.0001**
Time elapsed between the admission and the first prescription (hours)	10 (std 246.9)	11 (std 316.2)	7 (std 32.1)	0.435
*Clinical: State at the moment of the MR:				
Speech disorder				
Yes	256 (3.6%)	188 (4.3%)	68 (2.4%)	**0.013**
No	2033 (28.2%)	1331 (30.5%)	702 (24.7%)	
Hearing disorder				
Yes	442 (6.1%)	298 (6.8%)	144 (5.1%)	0.639
No	1847 (25.7%)	1221 (28.0%)	626 (22.1%)	
Visual disturbance				
Yes	661 (9.2%)	459 (10.5%)	202 (7.1%)	0.053
No	1628 (22.6%)	1060 (24.3%)	568 (20.0%)	
Cognitive impairment				
Yes	912 (12.7%)	650 (14.9%)	262 (9.2%)	**< 0.0001**
No	1377 (19.1%)	869 (19.9%)	508 (17.9%)	
Swallow disorder				
Yes	486 (6.8%)	313 (7.2%)	173 (6.1%)	0.449
No	3757 (52.2%)	2349 (53.9%)	1408 (49.6%)	
Nasogastric tube or stoma				
Yes	17 (0.2%)	11 (0.3%)	6 (0.2%)	0.952
No	976 (13.6%)	654 (15.0%)	322 (11.3%)	
Charlson score	2 (std 2.8)	2 (std 2.8)	2 (std 2.8)	0.036
Medication: State of the last valid medication prescription at the moment of the MR:				
Number of prescribed drugs per following ATC drug class				
A	3 (std 2.2)	3 (std 2.2)	3 (std 2.2)	**< 0.0001**
B	3 (std 2.5)	3 (std 2.5)	3 (std 2.5)	**0.0004**
C	2 (std 1.8)	2 (std 1.8)	2 (std 1.9)	0.983
D	0 (std 0.3)	0 (std 0.3)	0 (std 0.3)	0.091
G	0 (std 0.5)	0 (std 0.5)	0 (std 0.5)	0.837
H	0 (std 0.7)	0 (std 0.6)	0 (std 0.7)	0.374
J	0 (std 0.8)	0 (std 0.9)	0 (std 0.8)	**0.0002**
L	0 (std 0.5)	0 (std 0.5)	0 (std 0.4)	0.677
M	0 (std 0.5)	0 (std 0.5)	0 (std 0.5)	0.817
N	3 (std 2.4)	3 (std 2.3)	3 (std 2.5)	0.509
P	0 (std 0.1)	0 (std 0.1)	0 (std 0.1)	0.316
R	0 (std 0.9)	0 (std 0.9)	0 (std 0.9)	0.737
S	0 (std 0.5)	0 (std 0.4)	0 (std 0.5)	0.607
V	0 (std 0.4)	0 (std 0.4)	0 (std 0.4)	**0.001**
No ATC	0 (std 0.4)	0 (std 0.5)	0 (std 0.4)	**< 0.0001**
Prescriber was identified and was an intern	3131 (43.5%)	2105 (48.3%)	1026 (36.1%)	**< 0.0001**
Prescriber was identified and was not an intern	3356 (46.6%)	1989 (45.6%)	1367 (48.2%)	0.037
Reconciliation: State at the moment of the admission:				
Number of days elapsed between the admission and the last MR for the patient	71 (std 254.5)	82 (std 276.4)	54 (std 215.6)	**< 0.0001**
Total number of MR for the patient	0 (std 0.5)	0 (std 0.5)	0 (std 0.5)	**< 0.0001**

Data are mean (STD) or n (%).

Significant values are in bold.

*Some category variables do not add up to 100% because they were not recorded in the patient’s record, so we convert each variable into two columns with the combinations [0, 1] = yes, [1, 0] = no, and [0, 0] = unknown.

To develop a comprehensive prediction tool for identifying patients at risk of medication errors, a total of 52 variables (Table 1) were extracted directly or indirectly (by calculation) from the patients’ records. These variables were chosen on the basis of the advice of the pharmacists and according to similar studies on medication reconciliation^2,16,22^. Table 1 shows the general characteristics of the included patients in this study. It also shows the difference in patient characteristics (regarding the 52 variables) between the positive and negative cohorts (MEs and no-MEs patients). The mean age in the positive and negative cohorts was 73 years. The ratio of men 46.2% and 45.3% was almost equal in the positive and negative cohorts. The same applies to the mean BMI, which was found to be almost equally in both cohorts, 24.8% and 24.2%. The proportion of patients living at home was 74.8% and 83.3% in the positive and negative cohorts compared to 0.2% and 0.4% living in an institution. In Table 1, characteristics were compared between ME and no-ME patients with Student *t* test for quantitative variables and chi-square test for qualitative variables. The p-value in Table 1 showed whether there was a significant difference between positive and negative cohorts, for each variable. Variables with a p-value < 0.05 can be partially considered more significant than others for the label prediction.

### Prediction tool

Four machine learning algorithms—logistic regression (LR), support vector machine (SVM), extreme gradient boosting (XGB), and voting classifier (VC)—were used to develop the prediction models. Python 3.9.6 was used for coding the algorithm, and the scikit-learn library^32^ was used for all the ML models, except for XGB, for which the xgboost library^33^ was used. VC is a machine learning model that trains on a set of base models and predicts an output (class) based on hard voting or soft voting criteria. Hard voting aggregates the outputs of each base model and predicts the class label with the highest number of votes, while soft voting aggregates the probabilities of each class label and predicts the class label with the highest average probability. In this study, the SVM, LR, XGB, and random forest models were used as base classifiers for the VC classifier with hard voting criteria. Here, the default settings of the machine learning algorithms were used.

### Validation

The proportion of patients with MR data in the database was used as the gold standard, to which the outcomes predicted by the prediction model were compared. To address the problem of overfitting and to determine whether the model is generalizable, internal validation was performed using fivefold stratified cross-validation for each prediction model.

To evaluate model performance, several widely recognized metrics, F1 score, precision, and recall, were selected to measure model performance for predicting patients at risk of medication errors. The F1 score is defined as the harmonic mean of precision and recall. Precision measures the proportion of examples classified as positive that are truly positive, whereas recall measures the proportion of actual positives that are identified correctly. The precision is defined by precision = tp/(tp + fp), and the recall (also known as sensitivity) is defined by recall = tp/(tp + fn), where tp, fp, and fn are the numbers of true positives, false positives, and false negatives, respectively. Further metrics for the statistical performance analysis included the area under the receiver operating characteristic curve (AUROC). The receiver operating characteristic curve describes the trade-off between the true positive rate (TPR), also known as the sensitivity/recall, and the false positive rate (FPR) at various threshold settings. The AUROC is a measure of model performance: the closer the value is to 1, the better the prediction performance. We also used the area under the precision‒recall curve (AUCPR), which provides a more accurate representation of the impact the model can have on the pharmacist’s work^34^, given that the dataset is imbalanced. The AUCPR describes the trade-off between the positive predictive value (also known as precision) and the TPR at various threshold settings.

**Table 2:** Comparison of the performances of different ML models on imbalanced datasets.

	Imbalanced dataset			
Metric	LR	SVM	XGB	VC
Recall	0.59	0.44	0.48	0.67
Precision	0.53	0.58	0.60	0.55
F1 score	0.56	0.50	0.54	0.60
AUROC	0.68	0.68	0.70	0.71
AUCPR	0.60	0.60	0.64	0.65

**Table 3:** Comparison of the performances of different ML models on a balanced dataset (training).

	Balanced dataset			
Metric	LR	SVM	XGB	VC
Recall	0.63	0.66	0.55	0.75
Precision	0.52	0.51	0.58	0.52
F1 score	0.57	0.58	0.56	0.61
AUROC	0.68	0.68	0.71	0.71
AUCPR	0.60	0.59	0.64	0.64

**Table 4:** Comparison of the performances of different ML models on balanced datasets (training and validation).

	Balanced dataset			
Metric	LR	SVM	XGB	VC
Recall	0.63	0.66	0.55	0.75
Precision	0.65	0.65	0.69	0.65
F1 score	0.64	0.65	0.61	0.70
AUROC	0.70	0.70	0.72	0.74
AUCPR	0.70	0.71	0.73	0.75

## Results

Over a period of 7 years, data were collected only for patients who were hospitalized at various hospital departments for at least 24 h. During this period, 12,604 patients were randomly selected to undergo medication reconciliations. Pharmacist intervention was recommended for 2,878 of the 12,604 patients, meaning that 22.8% of patients were at risk of at least one unintended discrepancy.

The results of the different prediction models are shown in Tables 2 and 3, and 4. Table 2 contains the results for the prediction models (LR, SVM, XGB, and VC) based on the original imbalanced dataset. Table 3 contains the corresponding results when the training dataset is resampled (under/oversampled by the ENN and ADASYN), Table 4 contains the corresponding results when the training dataset is resampled (under and oversampled by the ENN and ADASYN) and the validation dataset is undersampled.

**Fig. 1:**
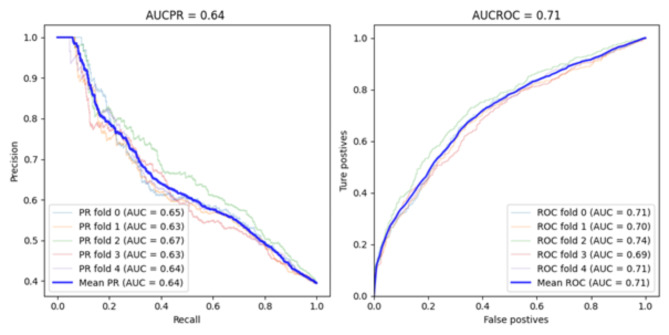
Accuracy of the VC model with a balanced training dataset: AUCPR and AUROC.

As with most healthcare datasets, one of the major hurdles when learning from imbalanced data is that the minority class is usually the class of interest. In the training set, having a class with few examples would cause any model to be skewed toward improper classification, as it will not receive enough examples for the class of interest. Therefore, balancing the dataset helps prevent the model from being biased toward the dominant class. Visual inspection of the values in Tables 2, 3 and 4 enables comparisons between the effectiveness of different prediction models. From the analysis of the different metrics used, it can be concluded that resampling the dataset leads to a viable improvement in prediction performance. The results in Tables 3 and 4 show that the prediction models (LR, SVM, XGB, and VC) produced higher recall, precision, F1 score, AUROC, and AUCPR values when the balanced dataset was used than when the imbalanced dataset was used, as shown in Table 2.

## Discussion

The main aim of this study was to improve patient safety by developing a tool that prioritizes admitted patients at risk of medication errors to undergo medication reconciliation by clinical pharmacists. To determine the extent to which the objectives underlying this study have been met, first, we will discuss the existing tool used at the CHU of Reims to select patients admitted for medication reconciliation and the extent of its effectiveness. We will later discuss the results obtained using our developed tool and compare them with the existing tool used to determine how efficient our tool is.

At our CHU of Reims, medication reconciliation is performed on a random sample of patients who have been hospitalized for at least 24 h and have had at least one prescription order. Here, we will discuss the results of the existing tool used at our CHU of Reims for selected patients for medication reconciliation in 2022. Out of 47,876 patients admitted to the hospital in 2022, only 2,037 (4.3%) were randomly selected for medication reconciliation. We found that medication reconciliation detected at least one unintended discrepancy in only 26% of the selected patients (536 of 2037 patients), leading to pharmacist intervention. These results can be read from two different points of view. On the one hand, the current procedure has succeeded in intercepting 26% of the positive patients who require pharmaceutical intervention. On the other hand, the presence of 26% of the patients within the selected random sample was considered high, which indicated that a large number of patients were not selected and may need pharmacist intervention. Due to limited resources and capabilities, the number of patients selected for medication reconciliation did not exceed 4.3% of the total patients admitted. Therefore, the presence of 74% of negative cases is considered a waste of the already limited resources that were supposed to be used to examine the files of other patients who may be at risk of medication errors.

The results in Table 2 show that the performance of the ML tools was affected by class imbalance, in which examples in training data belonging to one class greatly outnumber the examples in the other class. Two methods were used to balance the training dataset, allying a known undersampling method (ENN) and the oversampling method (ADASYN), to produce better defined class clusters. Our comparative results in Tables 2 and 3 show that using prediction tools with a balanced dataset provides more accurate results than does using an imbalanced dataset considering the recall metric.

**Table 5:** Ranking of prediction models based on Kendall W test results when used with imbalanced and balanced datasets.

Dataset	Models ranking	W
Imbalanced	VC > XGB > LR > SVM	0.491
Partial balanced	VC > XGB > LR > SVM	0.352
Balanced	VC > XGB > SVM > LR	0.422

Inspection of the results reported in Tables 2, 3 and 4 enables one to make comparisons between the effectiveness of the various models when used with imbalanced and balanced datasets. However, a more quantitative approach is possible using the Kendall *W* test of concordance^35^ to rank the effectiveness of all used models as shown in Table 5. This test was developed to quantify the level of agreement between a set of raters ranking the same set of objects. Here, we used this approach to rank the performance of the proposed models while used with imbalanced and balanced datasets. In the present context, the recall, precision, F1 score, AUROC and AUCPR metrics were used as the raters, and the LR, SVM, XGB and VC models were used as the ranked objects. The results of the Kendall analysis are reported in Table 5 and give the ranking for the various prediction models for each dataset. It is noted that the VC model were ranked first in all datasets. The effectiveness of prediction models is significantly impacted by using balanced datasets instead of imbalanced ones, as shown by the results in Tables 3 and 4. When a balanced training dataset is used (see Table 3), the improvement in the recall rate was 7%, 50%, 15%, and 12% for LR, SVM, XGB, and VC models, respectively, with a slight decrease in precision rate. The same results can be observed when the balanced training and validation dataset is used (see Table 4), but with an improvement in the precision rate of 23%, 12%, 15%, and 18% for the LR, SVM, XGB, and VC models, respectively, compared to the unbalanced dataset. Improvements in recall and precision rates led to improvements in the other related metrics, as demonstrated in Table 4. It is clear from these results (see Tables 3 and 4) that it is advisable to use balanced data to improve the effectiveness of prediction models. In what follows, we will discuss the results of our ML tools and, in particular, the VC model, as it achieved the best results. In what follows, we will discuss the results of the VC model when used with imbalanced and balanced datasets, where it achieved the best results among the other models (see Table 5).

Table 2 shows that the VC model performed the best among the other models for all metrics used (recall of 0.67, F1 score of 0.60, AUROC of 0.71 and AUCPR of 0.65) except for precision (0.55). When the balanced training dataset was utilized, the VC model also achieved the optimal outcomes, with a rise in recall rate to 0.75, despite no enhancement in the remaining metrics (see Table 3). Although the VC model showed a higher recall of 0.75, it showed an F1 score of 0.61. The F1 score was not as good as one might hope due to the low value of the precision metric. Low precision and high recall indicate that the class was detected well but also included observations of other classes (false positives), which can be confirmed by the AUROC curve results in Fig. 1. Table 4 shows the results of the prediction models when a balanced training and validation dataset was used. Table 4 shows a significant improvement for all models, with the best results (recall 0.75, precision 0.65, F1 score 0.70, AUROC score 0.74, and AUCPR 0.75) obtained by the VC model. Using balanced datasets (see Tables 3 and 4), the VC model achieved AUROC rates of 0.71 and 0.74, respectively. Generally, the AUROC results presented here are highly interesting, which means about 71% and 74% of the patients with MEs are correctly predicted by this model, and this is significantly better than chance.

**Fig. 2:**
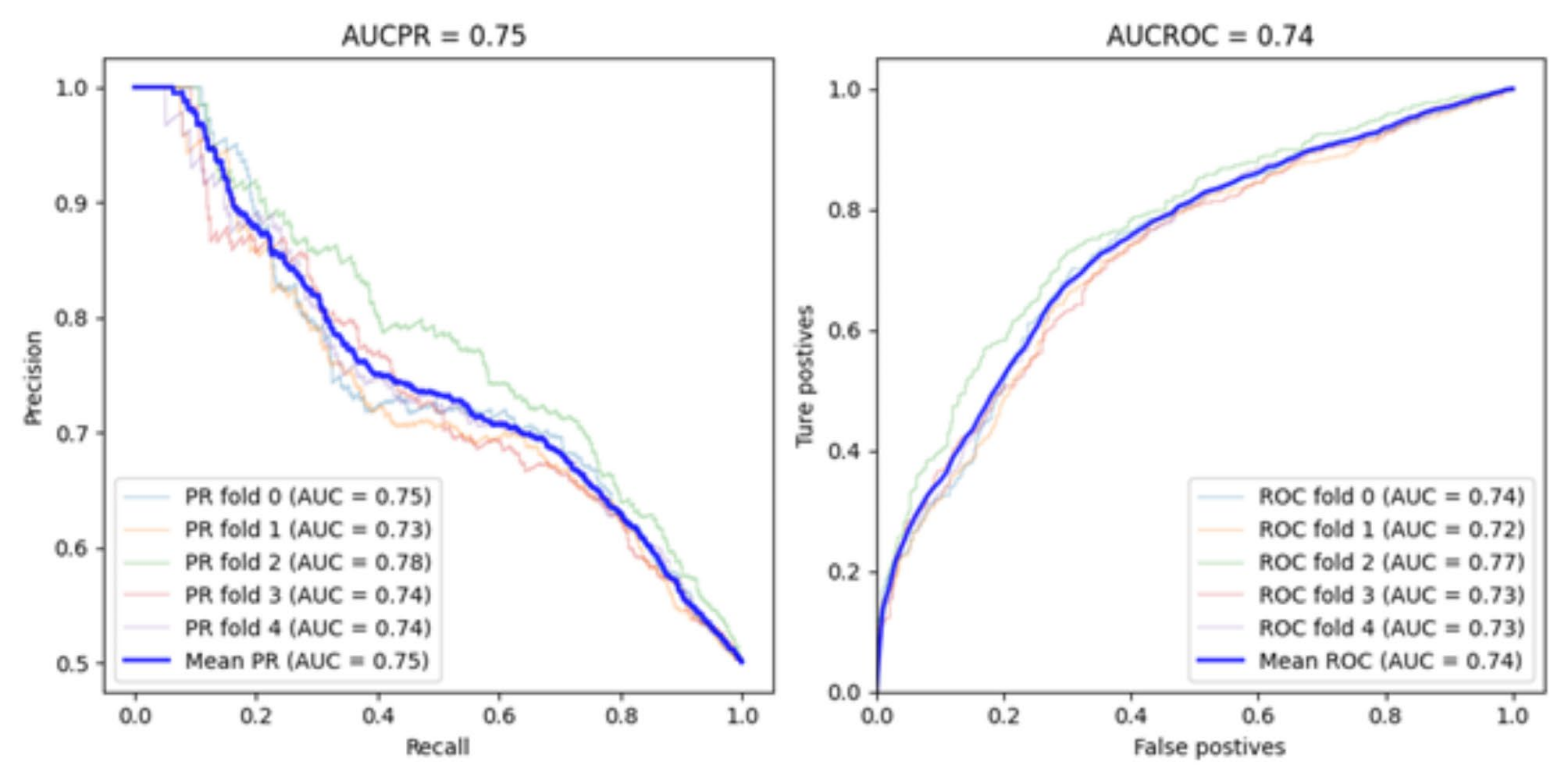
Accuracy of the VC model with balanced training and validation datasets: AUCPR and AUROC.

Due to the imbalance of the dataset used for validation, the AUROC can provide a false impression of the quality of the classifier (Table 2). As shown in Table 2, the AUROC was consider high 0.71, although the recall was 0.67, suggesting that the classifier performed well, which was not the case. However, although most of the negative cases are correctly classified, many of the positives may be misclassified, which is most important for model performance. We also noticed that in Table 3 despite the significant improvement in the recall metric, improvements in other metrics were not noticeable, especially in terms of precision, AUROC and AUCPR. The low precision is due to the increase in false positives, which can be confirmed by examining the AUROC curve in Fig. 1. The ROC curve plots the TPR versus FPR at different classification thresholds, and as the threshold decreases, the recall increases because we identify more patients who have medication errors. However, as our recall increases, our precision decreases because, in addition to increasing the number of true positives, we increase the number of false positives. Therefore, the AUCPR and precision were also used to evaluate the quality of the ML models. Thus, the recall and AUCPR values in Table 2 show that the performance of the ML model is not good, contrary to what the AUROC value reveals. To confirm the above results, we balanced the validation dataset using the undersampled ENN method such that it contained an equal number of positives and negatives, which improved all the metrics used (see Table 4). The results in Table 4 clearly revealed that the improvement in all the metrics was associated with an increase in the number of false positives because of the imbalance of the validation dataset. Figure 2 also shows significant improvement in the AUCPR compared to the AUCPR, as shown in Fig. 1.

Although the above results are very promising compared to those of existing tools, we still need to test our ML tool in a real-life evaluation to confirm its effectiveness. Therefore, to validate our tool, we performed a retrospective evaluation simulating real-life use to confirm its effectiveness. To conduct this evaluation, data from 317 patients who underwent medication reconciliation during the period 11/27/2023 to 02/04/2024 were used. Of the 317 patients, 93 were found to have at least one unintended discrepancy, which led to pharmacist intervention. Our ML tool was used to prioritize the 317 patients in descending order according to their likelihood of experiencing a medication error. A specific number of patient files (110 files) with high scores were subsequently selected. On the other hand, a group of clinical pharmacists was also recruited to carry out a selection process for the same number of patient files (110 files) but randomly. Finally, for each approach, the total number of patients who had at least one unintended discrepancy was recorded.

**Fig. 3:**
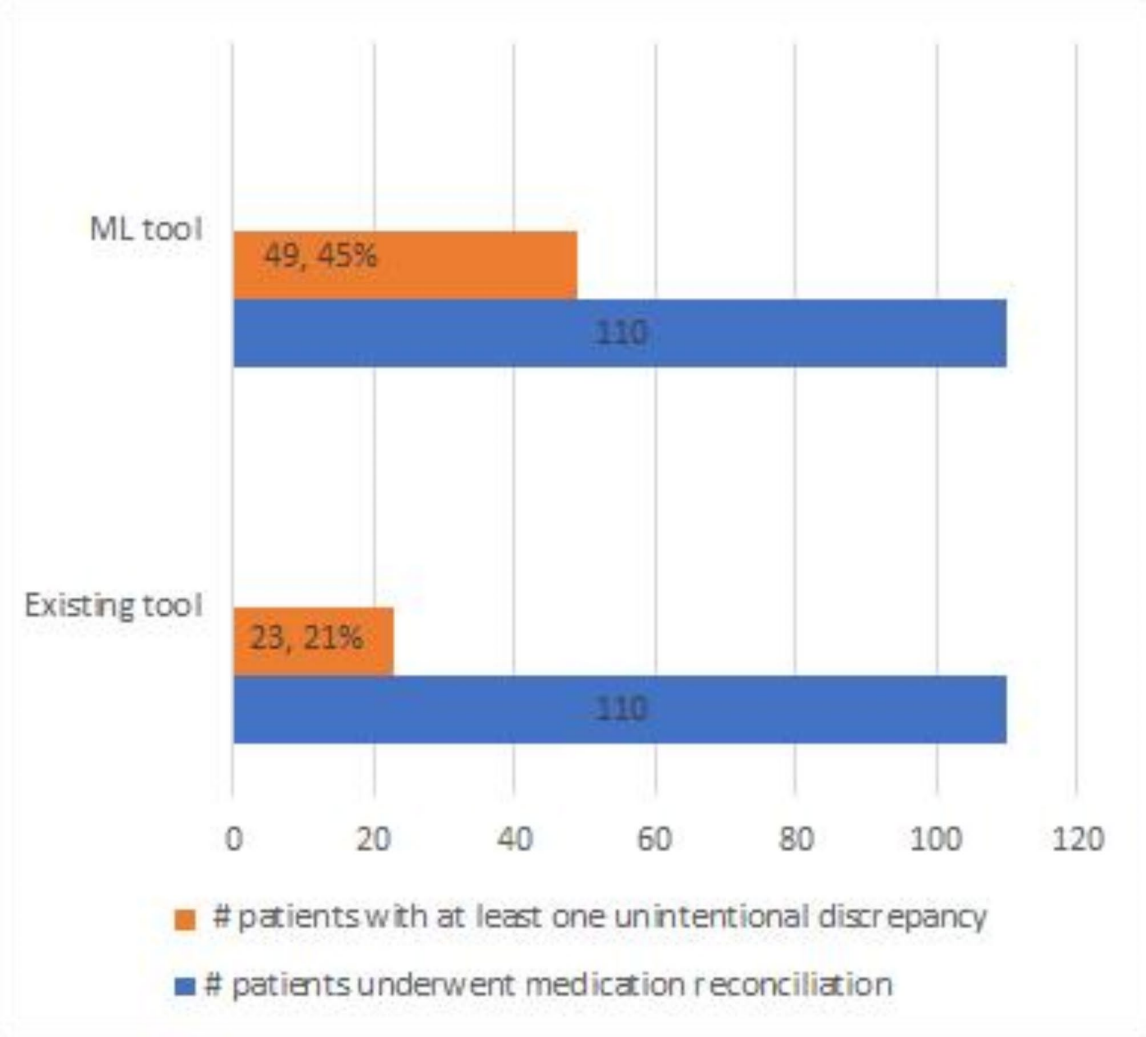
Comparison of medication reconciliation results using the ML tool (VC model) and the existing tool.

To determine the extent to which the objectives behind this study were met, first, we will discuss the results of the random approach used to select patients admitted for medication reconciliation and its effectiveness. Then, we will discuss the results obtained using our ML tool (VC model) for the same number of patients and compare them with those of the random approach. Figure 3 shows the results for both approaches. Using a random approach, 110 patients were randomly selected for medication reconciliation. Twenty-three of the 110 patients had at least one unintended discrepancy, while 87 patients did not. In other words, the random approach succeeded in identifying only 21% of the total selected patients. When using the ML tool, 110 patients were selected for medication reconciliation, and 45% of the total selected patients were found to have at least one unintended discrepancy, which led to pharmacist intervention, with an improvement of 113% compared to the random approach. These results confirm that the ML tool outperformed the existing tool used at CHU of Reims in terms of its ability to identify a greater number of patients exposed to medication errors. This approach could lead to increased capabilities of the clinical pharmacy department so that the medication reconciliation process would be more efficient and would reduce the burden on clinical pharmacists.

In summary, improving patient safety by reducing medication errors is a top priority in hospital health systems. Currently, medication reconciliation by clinical pharmacists is the standard method for preventing potential adverse drug events. These interventions are often limited by human resources, and patients at high risk for errors in their prescription orders need to be targeted. A strength of this study is that it included patients from internal medicine, surgical, and obstetric wards, as well as follow-up care and rehabilitation units; therefore, the findings are representative of daily clinical practice.

The single-center nature of our study was one of the limitations of this study. In addition, neonatology and intensive care unit patients were not included because these units do not use Easily software for medication prescription. However, due to the specificity of these units, pharmacists work directly on them. Therefore, medication reconciliations are never carried out in this type of services. Thus, there is no evidence of the accuracy of our model in identifying patients at high risk in these units; therefore, our model cannot be generalized to these patients. In fact, our study focused on medication reconciliation, which is mainly carried out in a hospital setting in the current medical practice. This focus limits the immediate applicability to primary care, where the data landscape and workflows differ significantly. In future work, we plan to incorporate data from other hospitals and possibly from primary care settings to improve the generalizability of our model. Our goal is to adapt the model to different healthcare environments, not just hospitals, by incorporating diverse data sources. While our current focus is on hospitalized patients, this work provides a solid foundation for expanding medication reconciliation efforts to other healthcare contexts, especially with a model trained on varied data sources.

It would be interesting in future work to improve the performance of our tool. The performance of an ML tool can be enhanced by obtaining abundant and accurate datasets. However, adding new and accurate data requires carrying out a large number of medication reviews, which is limited by a lack of well-trained human resources. This has motivated us to deploy our tool throughout other hospitals, as this will enable us to benefit from the expertise of a larger number of clinical pharmacists to confirm our findings and as an automated way to produce data that are used to update the model’s learning dataset.

## Conclusion

We developed and internally validated a machine learning-based predictive tool to predict patients with a high risk of at least one medication error during admission. Our predictive tool works as a digital tool to improve patient safety by identifying as many patients at risk of medication error as possible using available human resources. The positive experimental results of this tool showed a superior improvement of 113% over the existing tool by targeting patients at risk of medication errors upon admission to Reims University Hospital.

## Data Availability

The datasets used and/or analyzed during the current study are available from the corresponding author upon reasonable request.
